# Understanding the implementation of specialist maternity services for pregnant women with FGM/C in Germany: a situation analysis applying normalization process theory

**DOI:** 10.1186/s12978-026-02394-x

**Published:** 2026-07-03

**Authors:** Lisa Welcland, Marina A.S. Daniele, Isabel Runge, Lucia Rocca-Ihenacho, Christine McCourt

**Affiliations:** 1 Centre for Research in Maternal and Child Health, City St. George’s University of London, London, UK; 2 Hochschule für Technik und Wirtschaft des Saarlandes, Fakultät für Sozialwissenschaften, Saarbrücken, Germany; 3 Klinik für Frauenheilkunde, Universitätsklinikum Freiburg, Freiburg im Breisgau, Germany

**Keywords:** Female Genital Mutilation/Cutting, Midwifery, Maternity care, Birth Plan, Trauma-informed care, Normalization Process Theory

## Abstract

**Background:**

Female Genital Mutilation/Cutting (FGM/C) comprises all procedures involving the partial or total removal of the external female genitalia or any other injury to the female genital organs for non-medical reasons. There are about 600.000 women in Europe estimated to live with the consequences of FGM/C, of whom about 104.000 are residing in Germany. Less is known about maternity service provision for affected women living in Germany. An exploratory case study was conducted to examine the implementation of specialist services for women with FGM/C in a German Maternity Unit. This publication reports findings from a situation analysis exploring how specialist services were embedded into routine care.

**Methods:**

Data was collected using a mixed-methods design. A retrospective data analysis of clinical records from women with FGM/C who gave birth between 2020 and 2023 was conducted. Qualitative data provided insights of maternal health professionals experiences working within a specialist service for women with FGM/C. The two datasets were analyzed separately and then interpreted together using Normalization Process Theory. This approach was chosen to understand the underlying mechanisms involved in implementing a complex intervention inside a real-life context.

**Results:**

Results revealed that services were operationalized by using a trauma-informed approach adopted to individual needs and recorded inside women’s birth plans. Supportive mechanisms during the implementation were the presence of experienced colleagues, champions with sufficient position of authority in the service, an intrinsic motivation amongst staff, and accessible expert advice. Challenges were late midwifery contact, lack of time during consultations and fragmented postnatal care. Areas for further research could explore opportunities to address service fragmentation and focus on mechanisms to improve clinical coding.

**Conclusions:**

Specialist maternity services for women with FGM/C requires trauma-informed care, interprofessional collaboration and structural support. Systemic barriers such as fragmented care pathways, late access to midwifery care, and limited consultation time need to be further addressed to ensure equitable care in the long term. The best-practice model provides insights into the delivery of individualized maternity care for women affected by FGM/C and may inform the development of future models of care.

**Trial registration:**

DRKS00030704 (15.02.2024).

**Supplementary Information:**

The online version contains supplementary material available at 10.1186/s12978-026-02394-x.

## Plain english summary

Female Genital Mutilation/Cutting (FGM/C) involves the removal or injury of female genital organs for non-medical reasons. It can cause long-term health and emotional problems. In Europe around 600.000 women have experienced FGM/C, of whom about 104.000 live in Germany. We don’t know much about how maternity services in Germany address the needs of affected women.

This study looked into how a maternity unit in Germany provided specialist care for women with FGM/C. Medical records from 2020 to 2023 were analyzed and healthcare professionals were interviewed to learn about their experiences. Both sources of data were then combined and mapped to the domains of Normalisation Process Theory to better understand how maternity care for women with FGM/C was introduced in a service that is functioning well.

Findings revealed that the service was based on a trauma-informed approach. This means that care was adapted to women’s individual needs and included in their birth plans. Staff members valued the support of experienced colleagues, leadership from respected professionals, and access to expert advice. Additionally, the individual motivation to provide good care also played an important role. However, some challenges remained. These included late contact with midwives, limited time during consultations, and not seeing the same professionals after birth.

Overall, findings revealed that it is important that teams are well-organised and supported to provide sensitive care. Working to solve wider issues is important to make sure that care is safe, respectful, and treats everyone well in the future.

Understanding the Implementation of Specialist Maternity Services for Pregnant Women with FGM/C in Germany: A situation analysis applying Normalization Process Theory.

## Background

Female Genital Mutilation/Cutting (FGM/C) includes all procedures involving the partial or total removal of external female genitalia or any other injury of female genital organs for non-medical reasons [1]. Worldwide more than 230 million women and girls are affected by the practice with an estimated four million girls being at risk each year [2]. The procedure is associated with severe immediate and long-term health consequences. Immediate complications include excessive bleeding, infections, anaemia, urinary retention, wound healing complications, pain and genital tissue swelling [3–6]. Long-term effects persist throughout a woman’s life and manifest as physical, psychological and sexual health conditions. These include genital tissue scaring, chronic pain, menstrual problems, dyspareunia, recurrent urinary or reproductive tract infections [4]. An overview of FGM/C types is provided in Supplementary file 1.

Women with FGM/C face higher risks of obstetric complications: increase in prolonged labour, instrumental delivery, postpartum haemorrhage, perineal tears, episiotomy and caesarean section [3, 7]. Besides these risks, mental and sexual health consequences remain largely unrecognized during health service provision. A systematic review highlighted a strong association between FGM/C and depression, anxiety, somatization and Post-Traumatic-Stress-Disorder (PTSD) [8]. As research initiatives in Europe are still emerging, many affected women develop coping strategies and may often not actively seek mental health support, followed by missed diagnosis and gaps in specialist services [9].

In Europe 600.000 women are estimated to live with the consequences of FGM/C, including 103.947 residing in Germany [10–11]. To address these challenges, Germany ratified the Istanbul Convention on preventing and combating violence Against Women in 2017. The first progress evaluation urged German authorities to enforce data collection, implement mandatory trainings for health professionals, and integrate affected women’s perspectives into service development [12]. However, the extent to which recommendations have been implemented remains unclear, especially within maternity services.

Despite high-level policy efforts, gaps remain in the identification, documentation and clinical management of FGM/C within the healthcare system. Women with FGM/C face barriers in accessing specialized care, whilst healthcare providers feel unprepared to discuss or manage the condition. In 2017, a mixed-methods study on FGM/C assessed the situation in Germany [13]. Women reported mixed experiences with healthcare providers, with some professionals being informed and sensitive, while others did not initiate discussions about FGM/C. This left women uncertain whether it was due to a failure to recognize the condition or the discomfort in addressing it.

In 2023, a survey conducted in Germany explored the knowledge level and training needs among community gynaecologists, hospital-based physicians, midwives, and nurses [14]. Findings indicated that a lack of structural transfer regarding FGM/C within undergraduate medical curricula and specialist obstetrics and gynaecology training contributes to missed opportunities for care identification, resulting in incomplete documentation and coding within medical records. Interestingly, 80.7% had never attended training and only one-third were familiar with the four FGM types according to the WHO classification. Findings align with those of a scoping review on pre-service education and continuous professional development on FGM/C for maternal health professionals with countries from the Organisation for Economic Cooperation and Development, which found that training programmes are often described as limited and voluntary [15]. To date, no data are available on the impact of these factors on obstetric outcomes and service user experiences of care. This underlines the need for further research to address this evidence gap and generate knowledge to inform the development of quality-of-care indicators and strengthen outcome monitoring to ensure accountability.

Recognition of FGM/C is increasingly gaining attention in Germany, both at the national level and across individual federal states, as well as among NGOs and healthcare professionals. Despite growing awareness, there is a lack of empirical data on how maternity services address the needs of affected women. This knowledge gap is of concern given the increasing need for maternity services to respond to diverse populations shaped by global migration, asylum, and displacement, especially within the context of ongoing societal and political debates surrounding migration [16]. The WHO Global Action Plan on Refugee and Migrant Health (2024) emphasized the responsibility of healthcare systems to ensure continuity of care, equitable and accessible healthcare services, and to address chronic health conditions and cultural barriers experience by migrants and discplaced populations [17]. Reflecting on Germany’s history reinforces the ethical responsibility of healthcare professionals to critically engage with discrimination and inequities within healthcare systems and to ensure equitable and non-discriminatory maternity care. By investigating how health services in Germany address FGM/C, this study seeks to fill a knowledge gap and inform more equitable, evidence-based interdisciplinary approaches towards maternity service provision.

A single case study was conducted in a German Maternity Unit which offers a specialist service for women with FGM/C. The case study design comprised a situation analysis and a quality improvement project. This article reports on the situation analysis, which aimed to explore how specialist maternity care was implemented and sustained within routine care over time, and to use lessons learned to inform the development of future services in Germany.

Specifically, the objectives of the situation analysis were:

- To gain insights into the operationalization of the specialist FGM/C service;
- To understand the mechanisms and professional dynamics behind its implementation;
- To explore the resource mobilization and structural conditions that may influence its delivery in the long-term;

Therefore, Normalization Process Theory (NPT) was applied as a framework to synthesize findings from different data sources and to examine the underlying dynamics and contextual factors accompanying the implementation process [18]. Instead of focusing purely on outcomes, the use of NPT enables a deeper understanding of the individual and collective behaviours involved in service integration during routine practice.

## Method

The study was conducted in a large university hospital in Germany. This institution was identified following communication with an international expert in FGM/C based in Switzerland. Through this exchange, contact was established with gynaecologists specializing in FGM/C care in Germany. In the next step, the maternity unit was identified due to its activity in the field of FGM/C care provision. 

### Context

The German healthcare system is financed through statutory and private health insurance schemes. All residents are required to have insurance, with contributions to private health insurance (PHI) based on individual health, age and risk factors, while statutory health insurance (SHI) contributions are income-based. The SHI covers approximately 89% of the population with the remaining 11%, including civil servants and self-employed individuals, being covered by PHI [19].

Governance structures are decentralized with shared decision-making powers between state-, federal- and self-governance bodies. The Federal Joint Committee (Gemeinsamer Bundesausschuss, G-BA) determines which services are covered and the quality standards. Fragmented data systems and competing stakeholder priorities limit the availability of reliable national health data [20] and contribute to gaps in the visibility of FGM/C-related health needs.

In Germany every pregnant woman is entitled to antenatal care, which can be conducted by obstetrician/gynaecologists or (less often) midwives. A total of 692.989 live-births were recorded in 2023, including an out-of-hospital birth rate of 1,98% [21–22]. Women planning birth in hospital have their care transferred from community gynaecologists to the facility during the third trimester. As part of the transfer process, **w**omen attend an outpatient birth planning consultation, often with midwives (Supplementary file 2).

### Intervention

The Centre for Women with Female Genital Cutting was established in 2018 within the Women’s Clinic of a University Hospital. Initial leadership was provided by a senior physician who left the hospital in 2023 to specialize in reconstructive surgery. From 2020 onward, the clinic was supported by an additional gynaecologist with expertise in FGM/C-related care.

Since 2023, the Specialist Clinic has received funding from the Central Contact Point for Women with FGM/C, a federal project that provides accessible health and psychosocial services for affected women. This funding enabled the employment of a nurse during weekly clinics and covered translation services and patient transportation costs, when necessary.

The clinic offers FGM/C assessments, counselling and medical management of associated health complaints or complications. Specialist services include birth planning consultations and surgical interventions such as defibulation, scar tissue removal and reconstructive procedures during workshops with experts.

### Study design

A mixed-methods approach using a convergent-parallel design was used [23]. This design included a retrospective data analysis of case records from women with FGM/C who gave birth in the maternity unit and qualitative data that captured professionals’ experiences working within the service. The two datasets were analyzed separately and then interpreted together using Normalization Process Theory (NPT). NPT constructs represent four key stages of the implementation process: (1) Understanding the importance and value of implementing specialist services (coherence), (2) fostering professionals engagement and commitment to implementation (cognitive participation), (3) integrating services into routine clinical practice (collective action) and (4) evaluating lessons learned and the long term service sustainability (reflexive monitoring).

The quality improvement project will focus on establishing an interdisciplinary working group to reflect on the findings and develop a structured response through a participatory approach. The final evaluation of the case study should provide a basis for expanding future research from a single-case to a multiple-case study design [24].

**Fig. 1:**
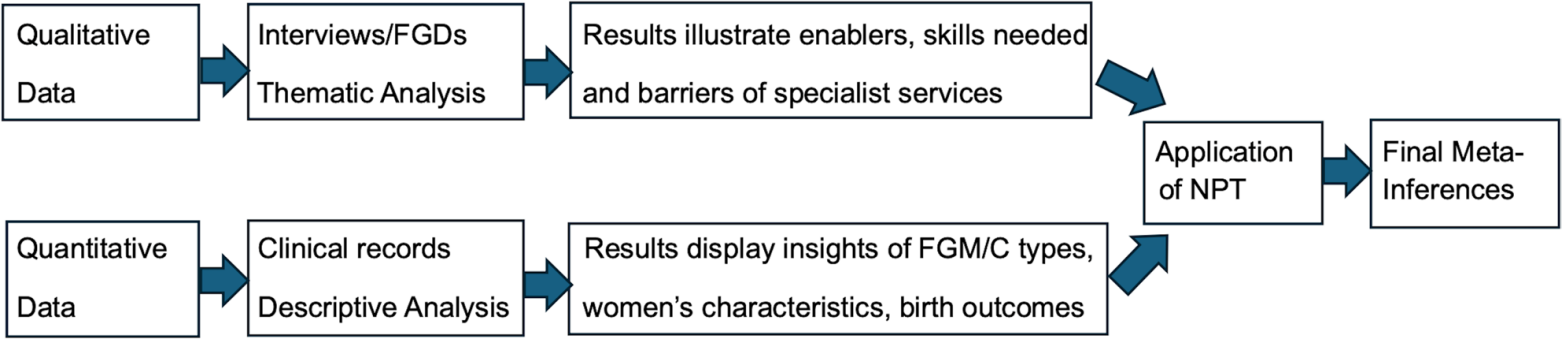
Overview of the mixed-methods convergent design

The Standards for Quality Improvement Reporting Excellence (SQUIRE) were applied to report the findings from the situation analysis [25] (Fig. 1).

### Data collection

Data collection was conducted during two field visits in April and June 2024. It included two sources: (1) Qualitative data from interviews and focus group discussions with healthcare professionals (Supplementary files 3 and 4) and (2) a retrospective review of clinical records from pregnant women with FGM/C.

(1) *Qualitative data:* The following healthcare professionals employed in the hospital were included: midwives, midwifery students, nurses, gynaecologists and paediatricians. Before the first field visit took place, information material about the study was displayed in the labour ward. The study was also introduced during team meetings and directly to professionals during field visits. Participants were recruited through convenience sampling and some by snowballing. A total of 21 participants were interviewed: gynaecologists (*n* = 3), midwives (*n* = 4), midwifery student (*n* = 1), nurses (*n* = 4), and paediatricians (*n* = 2). Additionally, one focus group was held with gynaecologists (*n* = 8). A second planned focus group with midwives could not be conducted due to scheduling constraints. However, data saturation was obtained during the second field visit, when no novel insights emerged from the interview data.

(2) *Review of clinical records*: To identify relevant cases, a request was sent to the hospital’s coding department to obtain an overview of FGM/C cases documented at the maternity unit between January 2020 and December 2023. Digital clinical records, including structured electronic documentation and free-text clinical notes, were reviewed. Relevant demographic, clinical, obstetric and service-related data, such as first presentation, gestational week, FGM/C classification, visits to specialist services, referral points, coded diagnoses and birth outcomes, were manually extracted into Excel spreadsheets. Clinical records were identified for 31 pregnant women with FGM/C who accessed services from the specialist clinic. Of these, 22 gave birth at the maternity unit. The documentation was rich and comprehensive, allowing first insights into women’s complex care needs related to FGM/C. Ongoing exchange with lead professionals from the FGM/C clinic further suggested that more FGM/C cases might have been seen without being coded.

### Data analysis and measures

Qualitative data and clinical records were first analyzed separately. Reflexive Thematic Analysis was applied to the qualitative data set. After an initial data familiarization, open and descriptive coding was conducted inductively by the first author to allow themes to emerge [26]. To support reflexive engagement and analytical interpretation, one interview and the focus group discussion transcripts were independently reviewed and coded by authors 2, 4, and 5, with findings being discussed during team meetings. The first author then continued independently with the thematic analysis and development of themes.

Due to low case numbers, the clinical case review was descriptive. Clinical data were organized into separate analytical datasets to explore women’s pathways through specialist maternity care and obstetric outcomes related to FGM/C. One analytical focus examined access to specialist services, referral pathways, and identification within the maternity care system. A second analytical focus explored obstetric management and birth outcomes among women who returned to the maternity unit for birth, including clinical complexities beyond FGM/C-related care needs.

In the next step, both the themes and the clinical case review findings were mapped onto the four NPT constructs using a more deductive approach through discussion with authors 2, 4 and 5. This approach allowed a structural exploration of how individual and collective behaviours contributed to service implementation [27].

### Reflexivity and rigour

The first author is a German-trained, white midwife with experience working as an HIV specialist midwife in the United Kingdom and as as a continuity of care midwife in Germany. Previous work with women with complex care needs informed a heightened sensitivity to issues relevant to FGM/C care, including continuity of care, stigma, interdisciplinary collaboration, and access to specialist services. The first author is also actively involved in midwifery education and the ongoing academization of the profession in Germany, providing insight into contemporary structural and professional developments within maternity care. This professional background positioned the researcher as both an insider and outsider throughout the research process, facilitating a contextual understanding of maternity service provision in Germany, while maintaining critical reflexivity.

Throughout the study reflexive field notes, regular supervision meetings, and interdisciplinary discussions informed ongoing critical reflection on data collection, analysis and interpretation of emerging findings. Participant involvement during the validation of findings prior to finalization contributed to reflexive practice and strengthened the trustworthiness of the interpretation of findings. Following data collection and analysis, preliminary findings were presented to participants during an online meeting to obtain their feedback.

### Ethical considerations

Ethical approval was obtained from a University Ethics Committee in Germany (Approval number: 23-1207_1-S1) and a University Ethics Committee in the United Kingdom (Approval number: ETH2324-1507).

Interviews were conducted after written consent was obtained, either in person or online, depending on individual preferences. All recordings were transcribed verbatim following the transcription guidelines by Kuckartz und Rädiker (2019) and participants were pseudonymized to protect confidentiality [28]. Transcripts were then imported into NVivo (Version 14, Lumivero 2023) for analysis.

## Results

**Fig. 2:**
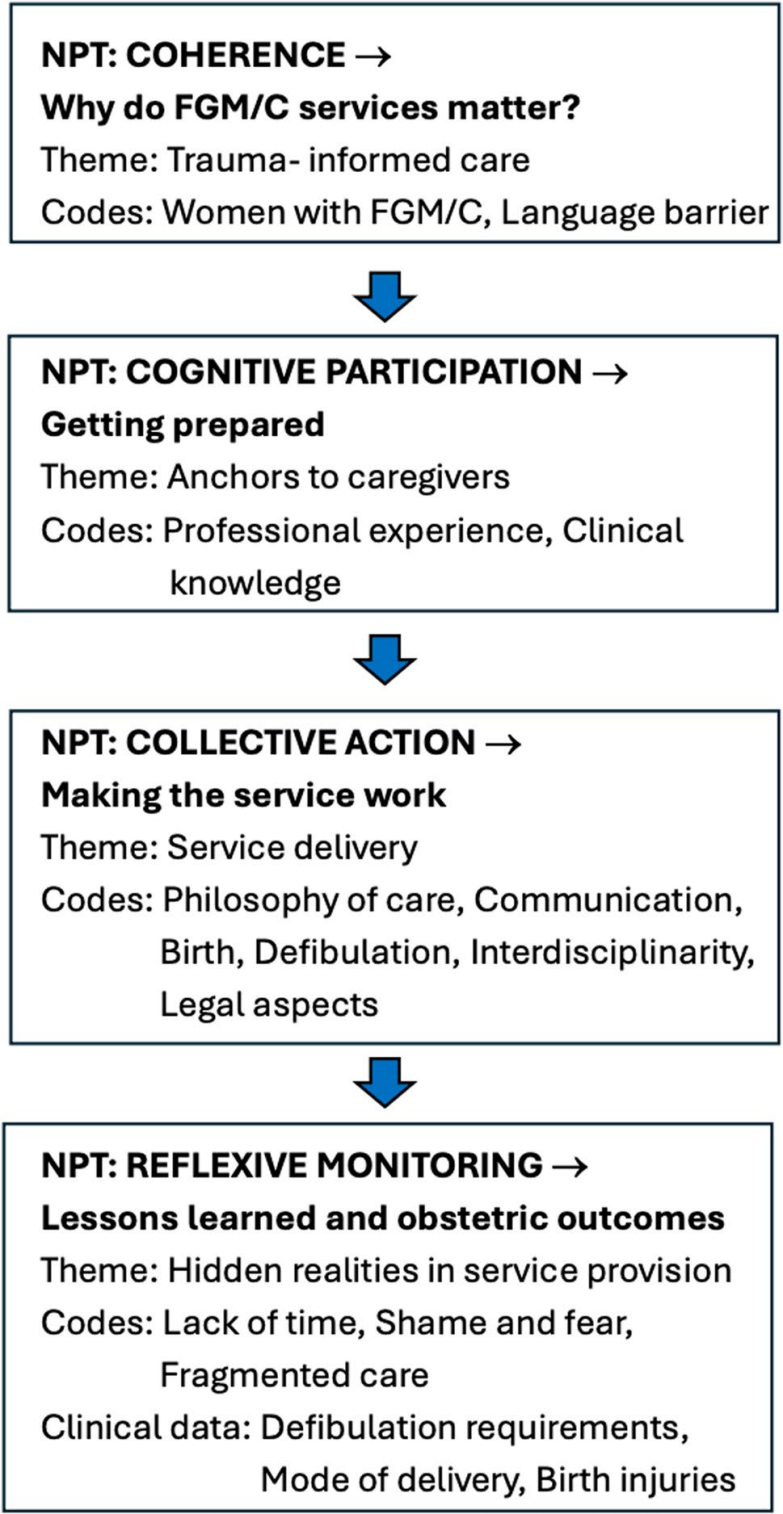
Alignment of codes, themes and clinical data with the NPT framework

Results are presented according to the four constructs of NPT. Figure 2 provides an overview of the alignment of inductively developed codes and themes with the NPT framework and illustrates the integration of quantitative data under the reflexive monitoring construct. While qualitative data informed all four constructs, quantitative data contributed to reflection on lessons learned, current service provision, and ongoing challenges relevant to sustaining specialist FGM/C services within routine maternity care.

### Coherence: why do FGM/C services matter?

Conversations with long-serving clinicians revealed that the specialist FGM/C service was initiated by gynaecologists, who recognized the urgent need of tailored care for affected women. Setting up the Centre for Women with Female Genital Cutting in 2018 marked the beginning of responding to their specific health needs. The philosophy behind services was developed around a trauma-informed approach, acknowledging that women with FGM/C may have experienced trauma and require safe maternity care to minimize potential triggers during childbirth.

Professionals recognized that the health needs of women with FGM/C were not comparable to those of women without FGM/C. They acknowledged these differences and emphasised the importance of considering individual experiences in order to provide tailored care. This shared understanding underpins the Theme “Trauma-Informed Care” and underlines why specialist services matter.

“Women who come to us have a lot of different experiences..ehm traumatic experiences..because it’s very often the case that women with a refugee background and a connection to FGM..that..ehm..they have also experienced forced marriage and violence..ehm..sexual violence.” Nurse 4, interview.

Setting up the FGM/C clinic established a safe space to discuss with women their specific health needs. Between January 2020 and December 2023 a total of 31 pregnant women with FGM/C attended the Maternity Unit. In addition to the diagnosis of FGM/C, 52% reported another health condition in their medical history (Table 1). Furthermore, 13% had a diagnosed mentHaving support mechanismsal health condition and 26% disclosed additional forms of violence alongside FGM/C. Women either self-referred or were referred directly by their community gynaecologists. Alternatively, they were sent through the National Office for Migration and Refugees, if the diagnosis was revealed as part of an asylum process.

**Table 1:** Characteristics, challenges and first presentation to maternity services

Category	Subcategory	Number (*n*)	Percentage (%)	Selection of expla-natory information
Health and social challenges	Pregnant women with FGM/C	31	100%	Total number of cases coded
	Additional health conditions	16	52%	Sexual Transmitted Infections, high BMI
	Diagnosed mental health condition	4	13%	Depression, severe psychological stress
	Disclosure of additional experience of violence	8	26%	Sexual, physical and/or emotional
First presentation	Hospital Birth Plan	10	32%	Gestational Diabetes, Intrauterine Growth Restriction (IUGR)
	FGM/C Specialist Clinic	11	35%	Scar-related pain, FGM-Certificate
	Other	10	32%	General emergency or inpatient services
Gestational week	< 12 weeks	4	13%	FGM/C-related complaints
	12 to 28 weeks	11	35%	Specialist Birth Plan
	> 28 weeks	16	52%	Routine Birth Plan
Referred by	Community Gynaecologist	23	74%	
	BAMF*	4	13%	
	Self-referral	4	13%	
Referred internally	Outpatient department to specialist services	6	19%	
	Inpatient department to specialist services	3	10%	
Distance to services	< 30 km	23	74%	
	30 to 100 km	6	19%	
	> 100 km	2	6%	
Known to services*	Due to FGM/C	12	39%	
	Other	3	10%	Previous pregnancy

BAMF*: Bundesministerium für Migration und Flüchtlinge (National Office for Migration and Refugees)

Field visits revealed the complexity of running specialist services and the challenges involved in integrating them into routine care inside a busy maternity unit. To reduce the number of missed appointments, women received an individual appointment reminder in advance. Before meeting the specialist gynaecologist, a comprehensive medical history was conducted either in German, English or French with support from the clinic nurse. For all other languages an interpreter was booked. Medical consultations usually last around one hour. If women attended with their daughters below the age of 18, consultation times increased to approximately 1.5 h to allow assessment and safeguarding discussions where appropriate.

The first specialist gynaecologists inspired others to start volunteering in the clinic. Over time the circle of volunteers grew further, and some nursing and midwifery students were able to obtain placements in the clinic. The exposure to FGM/C cases provided practical learning opportunities and had a strong emotional impact. One participant recalled her experience:

“The first time I volunteered, I was very touched afterwards, and I dealt with a lot..I thought about it..because I realized the women who came to us had differrent experiences..traumatic experiences.” Nurse 4, interview.

Healthcare professionals demonstrated awareness about FGM/C being a human rights violation. They were able to transfer this knowledge to the German context, including the importance of protecting children from the practice. Participants described how increased sensitization and knowledge supported conversations around prevention, and safeguarding as part of routine maternity care.

“So, when it comes to..that children should be protected from experiencing this..then you inform the parents about the medical risks of course..and that this can also be a life-threatening event and..ehm..all what it then means.” Gynaecologist 1, interview.

Moving from antenatal consultations to the birthing process itself, labour is often experienced as a painful journey and accompanied by fears or uncertainties. Midwives and gynaecologists working on the labour ward were aware of the importance of providing emotional stability and creating a safe environment, especially when women were giving birth in an unfamiliar setting. One gynaecologist reflected on the psychological challenges of birthing with FGM/C:

“I don’t imagine a birth to be particularly nice..psychologically alone…when I know that an opening is potentially necessary.” Gynaecologist 1, interview.

Participants expressed awareness about how migration, particularly amongst refugee women, affect health services. FGM/C was identified as part of it, and the importance of preparing maternity services for changing demands was underlined. One participant noted:

“With all the refugee women and so on, I believe that this (FGM/C) will be highly relevant for us in our future.” Student midwife, interview.

### Cognitive participation: Getting prepared

Whereas the coherence element illustrated the perceived need for specialist services, cognitive participation provided insights into how organizational structures were developed, and staff were prepared. From an organizational perspective, Germany’s Excellence Strategy played a vital role in setting up the specialist clinic to develop its profile in this area as a Cluster of Excellence. In addition, the Theme “Anchors to Caregivers” illustrated how role models, teamwork, training and defined responsibilities contributed to professional engagement and ownership. Interview participants discussed how clinicians can best prepare for encountering FGM/C during clinical practice. One recommendation was access to regular training through the institution and to differentiate between training levels:

“All staff working in the maternity unit here should have at least some basic knowledge about it.” Student midwife, interview.

Gynaecologists shared that they have regular access to FGM/C training at the Women’s Clinic. These were offered as short training sessions, either in person during the daily morning handovers or virtually, often scheduled in the afternoon or evening, when most of the routine work has been completed.

“Expert B and Expert C run regular training sessions and I watch them online.” Gynaecologist 2, interview.

Besides a basic knowledge about FGM/C and its impact on birth, it was also important that the labour ward team received specific training on how to care for women whose FGM/C status was not known antenatally. The preferred pathway included a prior birth planning appointment with the specialist clinic, as this supported shared decision-making based on the best available evidence and in partnership with the woman.

“I think if there are fears you can take them away. For example, I think with using a birth plan you can build up a lot of trust…if you decide it together and then there is perhaps the feeling that there are different alternatives or options.” Gynaecologist 5, Focus group.

Additionally, a personalized birth plan facilitated communication among professionals in the maternity unit, especially when women presented in labour and no experts were on duty. The plans were stored in the women’s digital clinical records and included information about birthing with FGM/C, whether a defibulation was required, and if so, a step-by-step guide supported the medical team to carry out the procedure.

Getting prepared for supporting women with FGM/C during birth, midwives and gynaecologists worked closely together with a clear understanding of their roles and the allocation of respective skills. Surgical interventions, such as defibulation, were seen as the responsibility of the medical team, whilst midwives provided continuity of care and emotional support. One participant described the role of midwives as:

“Midwives are the persons of trust and stability during labour” Student midwife, interview.

Recognizing women’s needs and understanding the contributions of each profession in this context helped staff prepare for specialist service provision during routine care. One midwife provided insights into how this is reflected within clinical practice:

“It’s very clear that this [FGM/C] will be discussed during consultation and the medical team takes care over it. They’ve agreed among themselves who’s going to deal with it [defibulation] and who’s responsible when someone [with FGM/C] comes in. Ehm..and it’s clear that it’s the doctor’s job.” Midwife 3, interview.

Additionally, the presence of experienced colleagues played a key role in motivating staff towards participation. Role models served as anchors for professionals and created a supportive environment where team members felt comfortable asking questions and seeking guidance during clinical practice, whether directly on the ward or by phone. One gynaecologist shared:

“I felt very safe because my first case was directly with Expert A…and that’s just a luxury situation […] I never worried in this setting that I was doing something wrong or felt insecure because there were always at least two people I could call at any time…if I somehow had a little uncertainty or something wasn’t clarified.” Gynaecologist 3, Focus group.

### Collective action: making the service work

By illustrating how specialist services for women with FGM/C were delivered and coordinated, the Theme “Service delivery” provides insights into the day-to-day realities of clinical practice.

Participants shared during focus group discussions that FGM/C-related services were integrated through informal, functional collaboration within the team. When support is needed, staff often reach out to experts for advice. A gynaecologist described a situation of uncertainty and how it was resolved through a simple phone call:

“I once had someone for an appointment prior to surgery [defibulation].I thought “Oh my God. I don’t even understand half of the words, which are used here.”.Ehm.I don’t think you were around [to the expert], so I called Expert A or C…but then you called me back afterwards and said: Hi, we’ve actually already discussed everything with her. Don’t worry. We take care of it.” Gynaecologist 4, Focus group.

This illustrated how internal processes developed over time and were embedded in routine practices. Whilst the birth plan supported shared decision-making in advance, implementation during labour relies on the interdisciplinary team on duty. Although there was no formal policy guiding maternity care for women with FGM/C, practical mechanisms existed to ensure high-quality care. Accessing experts or relying on individualized birth plans, when no experts were onsite, reassured the gynaecologists and midwives on duty:

“I always felt good ehm.because you were allowed to call Expert A, and now also Expert B, every time someone was announced to come in. And there was almost always a consultation [specialist birth plan appointment] in the clinic before. So, the person is literally known to maternity services. “. Gynaecologist 3, interview.

Having support mechanisms in place allowed resident gynaecologists to practice in an environment that promoted learning without pressure. The presence of a senior consultant on site reassured junior doctors and enabled them to perceive defibulations as less scary and to welcome the opportunity to develop new skills.

“Well, I was close about to do it once [defibulation]..and then the women became a caesarean section..but I had already overcome my fears.” Gynaecologist 9, Focus group.

Midwives contributed to the implementation of specialist services for women with FGM/C during labour by building trust and providing continuity of care. By focusing on these relational aspects, they helped to foster a reciprocal sense of security amongst professionals. A gynaecologist described how their continuous presence reassured not only the women, but also the medical team itself:

“The midwife is the more intimate caregiver. So, if things go well.I’m not actually there or I just say “hello” every two or three hours or in cases, where I am needed.” Gynaecologist 3, interview.

This collaboration between midwives and gynaecologists reflected the key mechanisms of collective action. Through trust-building, reciprocal reassurance and a clear allocation of responsibilities both professions contributed to the provision of safe care for women with FGM/C during labour.

Timely clinical feedback from gynaecologists also played an important role in strengthening midwives’ confidence when managing births with FGM/C. As one midwife explained:

“As soon as I get the medical go, whether the birth is affected by it [FGM/C] or not..it all depends on what was done…because if the labia were removed..then it’s traumatic for the woman..but it doesn’t affect how I deliver the baby.” Midwife 1, interview.

Over time, professionals developed informal referral systems, supported by increased awareness of FGM/C and the availability of specialist services. If women’s FGM/C diagnosis became known during an admission to the maternity unit, staff arranged for an expert consultation during their stay. These informal pathways illustrated how staff took ownership and actively engaged with specialist services as part of their routine clinical practice.

### Reflexive monitoring

This section brings together participants’ reflections on the implementation and delivery of specialist maternity services for women affected by FGM/C with clinical data from the maternity unit. By combining both data sources, it was possible to identify lessons learned, ongoing challenges, and areas requiring further attention to support service development and sustainability.

1. Hidden realities in service provision

The Theme *“Hidden realities in service provision”* came up as a theme during interviews and focus group discussions offering insights into challenges of providing maternity care for women with FGM/C. Participants used this opportunity to reflect on routine care practices and shared situations where they felt discomfort, shame or systemic pressure.

Midwives described challenges in relationship building with women affected by FGM/C. They pointed to barriers that extended beyond communication and reflected on how women’s experiences and emotional insecurities often became apparent during labour. These aspects were rarely addressed openly but their presence was felt behind closed labour ward doors:

“I often experience that we can’t get close to the women. So, there is the language barrier, but then..I don’t know..it seems there’s another barrier as well…and I think that’s something we often just ignore in our stressful everyday working lives.” Midwife 4, interview.

Gynaecologists reported similar challenges, particularly the lack of time to fully engage with women affected by FGM/C during clinical consultations.

“I have the feeling that often…there is just not enough time for this kind of conversations. So, if you see the woman randomly in another consultation, then you would actually have to say: Stop! Now I’ve made this diagnosis and now I would actually need one hour to talk to this woman…But.there is simply not that amount of time.” Gynaecologist 10, Focus group.

Participants also described feelings of shame and fear that made it difficult to speak about FGM/C. Past experiences or upcoming emotions hindered their ability to raise the topic or initiate a deep conversation. This kind of discomfort reduced opportunities to integrate FGM/C into maternity care as well as the likelihood of detection or appropriate documentation. One midwife shared:

“I was really ashamed..to talk about it…It was actually very difficult for me.” Midwife 2, interview

Beyond emotional barriers, structural limitations also hinder relationship building based on trust. Midwifery care often began late and was delivered under time pressure. Whilst basic care was provided, it was not always possible to tailor care to individual needs, leaving little opportunity to build trust or understand women’s personal histories. One midwife reflected on her work.

“We just see the women giving birth and then they are gone again.” Midwife 4, interview.

After birth women were transferred to the postnatal ward. General and paediatric nurses were the main caregivers there with midwives only being involved to a limited extent. The handover from the labour ward team often resulted in a fragmentation of care, where a new team, which was often unfamiliar with the woman, took over. One nurse shared insights from her work:

“We always say it during our handover and I think that when we hear the term FGM…each of us opens both ears and wants to know with what we have to deal now.How did the birth go? Which kind of birth injuries exist? How is her pain? What needs to be done?” Nurse 3, interview.

All participants valued specialist services but recognized the absence of feedback from service users. Without a mechanism to hear directly from women with FGM/C about their lived experiences during maternity care, their perspectives remain absent in the evaluation of care. One participant expressed a strong desire to learn more from women affected by FGM/C:

“I would like to learn from women who have experienced something like this and also experienced a birth. Somehow, I would like to know more about how they feel afterwards. How do you feel about it? How were you treated? And how did you generally experience it yourself.” Student midwife, interview.

Besides participants’ reflections on areas for continued learning and development, obstetric data were analysed to provide further insight into birth outcomes among women affected by FGM/C.

2. Reviewing obstetric data

Findings from the clinical records review provided insights into birth outcomes among women attending the maternity unit. Examining the need for defibulation, mode of birth, and birth injuries contributed to a broader understanding of maternity care provision and informed discussions with healthcare professionals about current care practices within the specialist service.

2.1 Defibulation

Data for 31 pregnant women affected by FGM/C were available. Three of them were already known to the service due to previous pregnancies with FGM/C and were therefore recorded as “known FGM/C cases”. As the defibulation was conducted in the past, these cases were not reassigned to a more specific FGM/C type. However, they were still included to ensure completeness of the clinical data. Of the remaining 28 women, 22 chose to deliver in the maternity unit, and nine of these were identified as requiring defibulation during specialist consultations (Table 2). In total, seven of the nine women identified received the intervention. In one case labour progressed too quickly and the other case required an emergency caesarean due to fetal distress. Reviewing the data showed that the need for defibulation was not exclusively associated with FGM/C Type III. Although most women requiring the intervention were classified with FGM/C Type III, one woman with FGM/C Type IIb was also identified requiring defibulation, emphasising the importance of individual assessment.

**Table 2:** Overview of FGM/C types and defibulation needs for labour

FGM/C Type	Number (*n*)	Percentage (%)	Defibulation		
			Planned	Performed	Timing
Type I	2	6%	N/A	N/A	N/A
Type IIa	2	6%	N/A	N/A	N/A
Type IIb	11	35%	1	0	First stage of labour (1)
Type IIIa	7	23%	5	5	Pregnancy (2) First stage of labour (3)
Type IIIb	4	13%	3	2	First stage of labour (1)
Type IV	2	6%	N/A	N/A	N/A
Known FGM/C	3	10%	unknown	2	unknown
Total	31	100%	9	9	7

2.2 Mode of delivery

Reviewing the 22 coded cases of women birthing with FGM/C, 11 were vaginal births and 11 were caesarean sections (Table 3). No case with FGM/C Type I was identified.

**Table 3:** Birth outcomes for women with FGM/C presenting for labour:

FGM/C Type	Number (*n*)	Spontaneous delivery	Instrumental delivery	Elective Caesarean	Emergency Caesarean
FGM/C Type II	10	3	2	2	3
FGM/C Type III	8	1	2	1	4
FGM/C Type IV	1	0	0	0	1
Known FGM/C	3	2	1	0	0
Total	**22**	**6**	**5**	**3**	**8**

From the vaginal deliveries, six cases delivered spontaneously. This group included two women with known FGM/C from previous pregnancies. The remaining five vaginal births were instrumental deliveries with one woman also known from a previous pregnancy. Among the 11 caesarean sections, three were elective and eight were performed as emergency procedures. The overall hospital caesarean rate in 2024 was approximately 37%. Besides FGM/C, women were coded with further birth complications, which were mainly failure to progress, green amniotic fluid and/or fetal distress. It remained unclear to what extent the observed complications were related to FGM/C, as additional risk factors and comorbidities were present. For example, six women had gestational diabetes, of whom two were managed on diet and four required insulin. Reflecting on these findings, participants highlighted that birth outcomes were influenced by multiple clinical and individual factors and emphasised the importance of considering FGM/C within a wider clinical context.

2.3. Birth injuries

Reviewing the 11 case records of women with vaginal deliveries, birth injuries could only be associated with FGM/C Type II and III as no other data regarding FGM/C Type I and IV were available (Table 4). There was only one case with no recorded birth injury. Nine cases involved minor perineal tears, and no third- or fourth-degree tears were documented. An episiotomy was performed in three cases. Additional vaginal tears occurred in three cases, and one case had a clitoral tear. Participants reflected that the absence of third- and fourth-degree perineal tears was an encouraging finding, although the small sample size limited interpretation.

**Table 4:** Birth injuries from women with FGM/C Type II, III and known FGM/C

FGM/C Type	Vaginal deliveries	No birth injuries	Perineal tear	Episio-tomy	Other birth injuries
FGM/C Type II	5	1	3	1	Vaginal tear (1) Clitoris tear (1)
FGM/C Type III	3	0	3	2	Vaginal tear (1)
Known FGM/C	3	0	3	0	Vaginal tear (1)

## Discussion

### Summary of findings

This situation analysis identified the following five key findings: (1) The service was operationalized through individualized and trauma-informed care practices, which included the use of birth plans, referral pathways and clearly defined responsibilities between midwives and gynaecologists. (2) Implementation was supported by informal mechanisms such as experienced colleagues who acted as authoritative champions, intrinsic staff motivation and accessible expert advice. (3) Professional dynamics were shaped by role models who fostered learning and by interprofessional collaboration. (4) Structural conditions supporting service delivery included the use of digital case records containing individualised care information, while limited consultation time, delayed midwifery contact and fragmented postnatal care were identified as barriers to sustain services on a long term. (5) Continuity of care emerged as an area requiring further attention, with relationship-building being essential when caring for women who may have experienced trauma.

### Understanding service implementation through NPT

Applying NPT provided insights into how specialist services were embedded within routine maternity care. Findings suggested that implementation was shaped by healthcare professionals who recognized unmet needs among women affected by FGM/C and developed ways to address them within existing service structures. The framework helped to understand the social and organisational processes supporting the integration of specialist maternity care into routine practice.

An important aspect of these processes was the development of trust and collaboration between professional groups. A study from the Netherlands applying NPT to midwife-led continuity of care models reported similar findings [29].The study identified trust as an essential requirement between professional groups when implementing new models of care. It was highlighted that trust between community and hospital-based professionals developed gradually through ongoing collaboration and growing confidence in each other’s competencies. This is in line with findings from the situation analysis, where role models, specialist support and interprofessional collaboration enabled midwives and gynaecologists to learn from each other and develop confidence in providing specialist care. At the same time, findings from the situation analysis suggested that strengthening collaboration across community and hospital settings was an important area for further development, particularly in relation to continuity of care and relationship-building.

Applying NPT underlined the importance of the wider organisational context in supporting implementation and sustainability. This aligns with the concept of the organisational space [30], which refers to structural and relational conditions enabling innovations to become embedded and sustained within routine care. Findings from the situation analysis reflected on the existence of such an organisational space through the availability of specialist support, regular access to training opportunities and interdisciplinary collaboration enabling specialist maternity care on the long-term. Previous research has shown that sustaining services depends not only on the commitment of healthcare professionals, but also on the organisation’s ability to create and maintain conditions that support collaboration, and service development [30].

### Operationalizing specialist services

The situation analysis revealed that the availability of specialist expertise alone was not sufficient to integrate services into routine maternity care. Instead, the challenge was to translate specialist knowledge into everyday clinical practice in a way that enabled healthcare professionals to access, share and apply this knowledge when caring for women affected by FGM/C. This was reflected in the theme “Anchors for Caregivers”, where experienced colleagues, specialist support, and opportunities for learning facilitated the operationalization of services.

One important aspect of care were the birth planning appointments. From a clinical perspective, these consultations created opportunities for individualised care and shared decision-making, placing women and their preferences at the centre. In addition, birth plans were an important communication tool between specialist and routine care by translating expert recommendations into clinical practice. The digital accessibility of birth plans inside the clinical records ensured that information regarding birth wishes, care needs, and defibulation requirements remained accessible throughout the maternity pathway. Similar findings have been reported in a research applying NPT into discharge planning. The study revealed the importance of communication tools and information-sharing systems in supporting coordination between healthcare professionals and maintaining continuity across different stages of care [31]. Therefore, birth plans functioned not only as a clinical document but also as a mechanism linking specialist and routine care with each other and ensuring that individual care needs remained visible beyond the specialist consultation.

In addition, the situation analysis emphasised the importance of clearly defined professional roles. Midwives and gynaecologists demonstrated a shared understanding of their respective responsibilities. Whereas midwifery care focused on providing continuity of care and emotional support during labour, gynaecologists were responsible for clinical decision-making and performing defibulation when required. Similar findings were described in a study from the Netherlands, where trust was a prerequisite for negotiating professional responsibilities when operationalizing a new service [29]. Interestingly, trust was reflected in the confidence healthcare professionals placed in each other’s competencies and in the respective allocation of skills. In the situation analysis, trust was fostered through the presence of clinical experts who acted as role models and created safe learning environments in which staff could seek advice, develop skills and gain confidence in caring for women affected by FGM/C. This trust was reflected not only in the availability of specialist expertise when required, but also in the confidence midwives and gynaecologists placed in each other’s competencies and expertise.

### Learning from obstetric data

Reviewing the clinical data provided insights into the complex care needs that accompanied FGM/C and influenced maternity care planning, including medical, psychosocial, and social circumstances documented within the records. Findings suggested that women should not be understood through their FGM/C status alone. The data provided insights into the presence of additional pregnancy-related complications and comorbidities, which may have contributed to the observed caesarean section rate that was higher than reported in other European studies [32, 33]. Similar findings were reported among Somali-born women in Norway and a review of migrant women in high-income countries suggested that such outcomes may also reflect broader factors associated with migration and displacement [32, 34]. Given the small sample size and descriptive nature of the clinical data, findings should be interpreted cautiously. However, the data underlined the importance of holistic maternity care approaches recognising the broader social, psychological, and health-related factors affecting women with FGM/C.

Another lesson learned refers to the management of defibulation within routine maternity care. Findings suggested that women requiring defibulation could be supported through individualised care planning and specialist care, with the timing of the procedure tailored to women’s preferences and clinical circumstances. This is in line with existing evidence showing no significant differences in obstetric outcomes between antenatal and intrapartum defibulation, supporting a woman-centred approach to decision-making [35–37]. An interesting finding was that the need for defibulation was not exclusively associated with FGM/C Type III, as one woman with FGM/C Type II also required the procedure. This highlighted the importance of individual assessments instead of relying purely on classification systems. Despite its clinical relevance, defibulation is often not routinely included within maternity training curricula [15, 38]. The situation analysis suggested that, within the specialist service, defibulation was successfully integrated into routine maternity care through training, supervision, and access to clinical expertise. Interestingly, previous research emphasised that advanced competencies in FGM/C care should be guided by skills and clinical expertise rather than professional titles or predefined professional roles [37].

#### Implications for practice and further research

Findings from the situation analysis highlighted several implications for the future development of specialist maternity services. One implication relates to continuity of care and relationship-building. Participants highlighted challenges in establishing trusting relationships with women, particularly when care remained fragmented across the continuum of care during pregnancy, birth and the postnatal period. This finding is supported by evidence from midwife continuity of care models, which have been associated with improved maternal and neonatal outcomes, lower intervention and caesarean section rates [39]. These benefits appeared to be relevant for women with complex social and clinical care needs, where continuity of care facilitates relationship-building, coordinated support, and personalised care across the maternity pathway.

Another practice implication referred to the further development of a trauma-informed care approach for women affected by FGM/C. Whilst healthcare professionals demonstrated an awareness of the potential impact of trauma on some women affected by FGM/C, the approach should not be categorised as a fully realised model of care. It rather reflects an ongoing process of recognising what women may have experienced and that these experiences can influence maternity care needs. Continuity of care across service boundaries should be strengthened and healthcare professionals supported in developing advanced skills in sensitive communication, relationship-building, and the prevention of potential triggers during labour and birth. Evidence from caseload midwifery models of care further suggested that sustained relationships provide healthcare professionals with the time to build trust, understand women’s individual circumstances, and identify social, psychological, or clinical needs that may not be disclosed during fragmented episodes of care [40]. In the German context, strengthening continuity of care and developing advanced competencies in communication, relationship-building, and trauma-informed care align with current developments related to the academisation of midwifery and the expansion of professional roles. However, the perspectives of women affected by FGM/C remain essential to ensure that future service developments reflect their needs and priorities.

### Strengths of the study

A key strength of this study is that it is grounded in clinical practice and provides insights into maternity care from a specialist service for women affected by FGM/C. To our knowledge, this is one of the first interdisciplinary research projects in Germany to be conducted collaboratively by an academic team, a midwife as principal investigator, and a gynaecologist as clinical lead within a maternity unit. The study contributes new insights into maternity care for women affected by FGM/C and addresses an important knowledge gap in the German context.

Existing evidence on FGM/C in the German healthcare system has so far focused either on the general experiences of women or healthcare professionals [13], on health consequences and treatment [41–42], or on practical guidance [43]. Using a mixed-methods design informed by NPT explored how specialist maternity services were implemented and embedded within routine care.

#### Limitations

The study was designed as a single case study, limiting the generalizability of findings to other maternity units, especially given that practices reflect a specialist service rather than routine maternity care. In addition, the possibility of selection bias should be acknowledged, as findings may reflect the perspectives of a particularly motivated staff group rather than the full range of views within the team.

A key limitation was the absence of service users’ perspectives. The findings reflect healthcare professionals’ experiences and clinical documentation, representing an institutional and provider-based view of maternity care. As women’s perspectives are essential for understanding the acceptability and quality of care, future research should explore the experiences of women affected by FGM/C, ideally through community-based approaches outside the hospital setting.

Regarding the quantitative data, the low number of coded FGM/C cases limited the analysis to descriptive statistics. Therefore, findings should be interpreted with caution, particularly when considering obstetric outcomes and their wider applicability beyond the study setting.

## Conclusions

This situation analysis provided insights into the implementation of a specialist maternity service for women affected by FGM/C. Applying Normalization Process Theory helped to understand how specialist care was embedded within routine maternity services and highlighted the organisational, professional, and contextual factors influencing implementation.

Findings suggest that specialist expertise, leadership, role modelling, interprofessional collaboration, and opportunities for shared learning supported the integration of FGM/C-related care into practice. At the same time, fragmented care pathways, limited continuity of care and restricted opportunities for relationship-building remained important challenges. The findings further point towards the relevance of trauma-informed and relationship-based approaches when caring for women affected by FGM/C.

As the situation analysis was based on healthcare professionals’ perspectives and the review of clinical records, the experiences and views of women affected by FGM/C remain an important area for future research. Understanding women’s perspectives on maternity care will be essential for informing the further development of responsive, equitable, and sustainable services in Germany.

## Supplementary Information

Additional file 1: Types of Female Genital Mutilation/Cutting.

Additional file 2: Organisation and transfer points of maternity care in Germany.

Additional file 3: Semi-structured Interview Guide.

Additional file 4: Interview Guide for Focus Groups.

## Data Availability

The datasets analyzed during the situation analysis are not publicly available due to the need to protect participants confidentiality. Data may be made available upon reasonable request from the corresponding author.

## Abbreviations

FGD: Focus Group Discussion
FGM/C: Female Genital Mutilation/Cutting
NPT: Normalization Process Theory
OECD: Organisation for Economic Co-operation and Development
PHI: Private Health Insurance
SHI: Statutory Health Insurance
WHO: World Health Organization
