# Supplementary material for: Understanding the implementation of specialist maternity services for pregnant women with FGM/C in Germany: a situation analysis applying normalization process theory
Source: Reprod Health. 2026 Jul 3;23:132. doi: 10.1186/s12978-026-02394-x (PMC13332614; doi:10.1186/s12978-026-02394-x)
Supplement: Supplementary file 1 — Additional file 1: Types of Female Genital Mutilation/Cutting. [file 12978_2026_2394_MOESM1_ESM.pdf]

Supplementary file 1: Types of Female Genital Mutilation/Cutting

Type I	Partial or total removal of the clitoris glans, clitoris hood or both
Type II	Partial or total removal of the clitoris glans, the labia minora with or without removal of the labia majora
Type IIa	Removal of the labia minora
Type IIb	Partial or total removal of clitoris hood/glans with removal of labia minora
Type IIc	Partial or total removal of clitoris hood/glans with removal of labia minora and. majora
Type III (Infibulation)	Narrowing of the vaginal opening with the creation of a covering seal through cutting and repositioning of labia minora or majora or both. Covering is done with or without removal of clitoris hood/glans.
Type IIIa	Removal of labia minora
Type IIIb	Removal of labia majora
Type IV	All other harmful procedures on female genitals for non-medical reasons
Defibulation	Medical intervention of cutting the sealed vaginal opening of women with FGM/C Type III (Infibulation).

Adapted from WHO (1997) Types of female genital mutilation. Types I and IV are not described in further detail, as only Types II and III occurred as documented subtypes within the study sample. Defibulation was included into the table as a relevant medical intervention due to its importance in the clinical management of women affected by FGM/C.
