# Supplementary material for: Understanding the implementation of specialist maternity services for pregnant women with FGM/C in Germany: a situation analysis applying normalization process theory
Source: Reprod Health. 2026 Jul 3;23:132. doi: 10.1186/s12978-026-02394-x (PMC13332614; doi:10.1186/s12978-026-02394-x)
Supplement: Supplementary file 2 — Additional file 2: Organisation and transfer points of maternity care in Germany. [file 12978_2026_2394_MOESM2_ESM.pdf]

MATERNITY CARE	ANTENATAL CARE	INTRAPARTUM CARE	POSTNATCAL CARE
ORGANISATION OF CARE	1. Obstetric care<br>2. Shared care: Obstetric and midwifery care<br>3. Midwifery-led care	1. Maternity care at the labour ward (midwife and obstetrician)<br>2. Midwifery-led care inside the labour ward<br>3. Midwifery-led care in a birth centre or at home **	1. Inpatient Postnatal Care: Nurses, Midwives, Obstetricians and Pediatricians<br>2. Outpatient Postnatal Care: Midwives, Obstetricians and Paediatricians
PLACE OF CARE *	Community	Hospital (1+2) and Community (3)	Hospital and Community
TRANSFER OF CARE	Outpatient booking appointments (1+2) for hospital births (approx. 34-36 GW). In case of high-risk pregnancies or specialised care earlier.<br><br>In case of arising risks and complications (3) transfer for obstetric consultation or permanent obstetric care.	In case of complications (1) transfer to routine labour ward care (2) or from the community to hospital services (3).	Routine discharge after approx. 48-72 hours back to community (1) with the second paediatric examination usually takes place at the postnatal ward. Early discharge (approx. 6-12 hours after SVD) possible on request.<br><br>Discharge from midwifery postnatal care at 12 weeks postpartum.

Supplementary file 3: Organisation and transfer points of maternity care in Germany

*: Maternity care at community level is entirely provided by freelance gynaecologsits/obstetricians and midwives. Hospital services vary, but the majority of care is provided by employed maternal health professionals. **: In Germany, approx. 2% of deliveries take place with midwifery-led care in a birth centre or at home. ***: The second paediatric examination is scheduled between postnatal day 3 and 10, which is often combined with the newborn screening (36-72 hours after birth).
