# Supplementary material for: Understanding the implementation of specialist maternity services for pregnant women with FGM/C in Germany: a situation analysis applying normalization process theory
Source: Reprod Health. 2026 Jul 3;23:132. doi: 10.1186/s12978-026-02394-x (PMC13332614; doi:10.1186/s12978-026-02394-x)
Supplement: Supplementary file 3 — Additional file 3: Semi-structured Interview Guide. [file 12978_2026_2394_MOESM3_ESM.pdf]

Semi-structured Interview-Guide:

1. **What has been your experience with caring for women with FGM?**
   Probes:
   - When was the first time you cared for a woman with FGM?
   - What can you remember from this experience?
   - Did you feel prepared?
   - If not, what would have been helpful?
   - Did you care change over time?
   - Which skills are required to care for a woman with FGM?

2. **Was FGM included into your training?**
   Probes:
   - If yes, how was it thought?
   - Did you learn about FGM with lectures or during your practical placements?
   - Where you equipped with all necessary skills to care for women with FGM?
   - Do you feel competent to care for women with FGM?

3. **How do you feel about asking a woman about FGM?**
   Probes:
   - Do you feel confident to ask a woman about FGM during your first patient contact?
   - Is there anything you need to ask the question?
   - Whom to, would you ask the question?
   - Is there anything you need from your environment?
   - Are there any exclusion criteria, why you would not ask the question?
   - Who would you handle the situation in case of a language barrier?

4. **When a woman disclosed FGM to you, how would you proceed?**
   Probes:
   - Would you ask for help?
   - Whom would you ask for help?
   - How would you classify the type of FGM?
   - Would you do an examination?
   - How would you document your findings?
   - Where would you document your findings?
   - Is there anything important to consider regarding the confidentiality of the woman?

5. **Is there anything else you might need to discuss with the women?**
   Probes:
   - How would her further clinical care pathway look like?
   - Are there legal requirements you would discuss?
   - If yes, what would they be?
   - Do you feel prepared for a situation like that?
   - Is there anything else you would need?

6. **If you reflect on your previous experience, how do you think experience women with FGM experience inside your service?**
   Probes:
   - What are the strengths of your service?
   - Where do you see challenges with your service provision?
   - How would you describe the decision-making process with woman?
   - What do you think need women with FGM for a positive birth experience?

7. **Are there areas you would like to deepen your knowledge?**
   Probes:
   - How confident to you feel to classify the various FGM types?
   - Did you receive training about the classification process?
   - If yes, how was the training arranged?
   - Could you practice the classification process in a skills lab?
   - Would skill training be an interesting training component for you?
   - In case of a future training activity, would you prefer monodisciplinary or interdisciplinary teaching?
   - Which training areas seem to be essential to you in order to become a confident practitioner?
   - In case of no financial restrictions, how would a perfect FGM training look to you?

8. **Would you feel confident to perform a deinfibulation?**
   Probes:
   - The deinfibulation falls into the responsibility of which profession?
   - Please explain your statement.
   - Where do you see the benefits of working in an interdisciplinary team?
   - Are there also challenges involved?
   - Can you say something about the care philosophy from your profession regarding the care for women with FGM?

9. **Is there anything else you would like to share?**
