# Supplementary material for: Understanding the implementation of specialist maternity services for pregnant women with FGM/C in Germany: a situation analysis applying normalization process theory
Source: Reprod Health. 2026 Jul 3;23:132. doi: 10.1186/s12978-026-02394-x (PMC13332614; doi:10.1186/s12978-026-02394-x)
Supplement: Supplementary file 4 — Additional file 4: Interview Guide for Focus Groups. [file 12978_2026_2394_MOESM4_ESM.pdf]

Semi-structured Guide for Focus Groups

1. **Was there any learning content on FGM as part of your training?**

Probes:
- If yes, which topics were discussed?
- How did you encounter the topic: During theoretical teaching or within clinical practice?
- How did you acquire your professional knowledge?
- Do you feel prepared to care for women with FGM?

2. **How do you feel about addressing FGM with a woman?**

Probes:
- How do you ask about FGM?
- When do you ask the question about FGM?
- Which internal and external factors influence your communication behaviour?
- Are there situations in which you would avoid raising FGM?
- How do you deal with the question in the event of a language barrier?

3. **How do you proceed when a woman discloses FGM?**

Probes:
- Would you call for help or support?
- If yes, who would you approach?
- Would you seek to confirm the type of FGM?
- If yes, what would you do?
- How would you document your findings?
- Is there anything else you would need to consider during documentation?

4. **Are there any other topics you would discuss with the woman?**

Probes:
- What do you know about her clinical care pathway after the identification of FGM?
- Are there any legal aspects to consider?
- If yes, what do you know?
- Do you feel prepared to have a discussion about safeguarding children with regards to FGM?
- If not, what would you need to have this conversation?

5. **When you reflect on your previous care for women with FGM: How do you think women experience your service?**

Probes:

- What is special about your service?
- Where do you see challenges or potential for improvement?
- How would you describe the decision-making process with affected woman? What is important for women with FGM to have a positive birth experience?

6. **In which areas of FGM would you like to acquire further knowledge?**

Probes:

- How confident do you feel in classifying the different types of FGM?
- Did you receive training on case identification?
- If yes, how was the training designed?
- Could you practice the classification process of FGM in a skills lab?
- What learning content about FGM would you like to deepen in the Skills lab?
- Would you prefer either monodisciplinary or interdisciplinary teaching on FGM?
- What do you need to feel confident in caring for women with FGM?
- How would a perfect training session on FGM look like?

7. **Would you feel confident to perform a deinfibulation?**

Probes:

- Deinfibulation is the responsibility of which profession?
- Please explain briefly why?
- Where are opportunities and challenges within interdisciplinary cooperation?
- How would you describe your professional philosophy when providing care for women with FGM?

8. **Is there anything else you would like to share with us?**
